# Breeding and Feeding

**Published:** 1887-01

**Authors:** A. S. Heath

**Affiliations:** Baldwins, Long Island, New York


### ART. VI.—BREEDING AND FEEDING.

BY A. S. HEATH, M. D.
*Baldwins, Long Island, New York.*

The first aim and object in the great art and science of breeding and feeding, of our domestic animals, is to secure and maintain a sound constitution, healthy and strong functions of digestion and assimilation. The next definite object should be to improve the essential powers in animals for a special purpose—either for milk, meat, butter, cheese, wool, beauty, speed, or labor—as a demanded product. Tyros in breeding and feeding, the world over, have squandered time and money in the beginning, by using too low a grade of animals to breed from. If animals as sires or dams had proven themselves as great animals were used in breeding establishments, too often their strength, stamina, and constitutions had been impaired by age, excessive use or abuse, to yield the results which might have been expected from them in a state of maturity and perfection. For the offspring inherits the sum of the qualities, characteristics, powers, aptitudes, stamina, strength, vigor, internal and external conformations and organizations peculiar to both parents. The modified combination in the offspring may be difficult at times to trace even by the best expert, yet the constituents of the compound animal is nevertheless inherent in the young. The greater prominence in the different offspring of the inherited characteristics of the same parents must depend, to a considerable degree, upon a change of condition at the times of begetting. But all things being alike, if possible, the nearer alike the compound qualities and characters of the parent must result We must not lose sight of the artificial part of the existence of the animals bred from. For, as in plant life, so in animal life there is a strong tendency to return to the natural state, as evinced by families of animals, or as apples to return to the original crab. Engrafted plants are always the same as the parent stock, while seedlings always differ. In breeding we do not engraft, but always use a compound seed to perpetuate the species.

The activity of the reproductive functions largely depend upon the healthy state of the nervous system, as well as upon the offices of nutrition and all the other animal functions hence the baneful influence of every cause that in any degree impairs the nervous energies, or weakens the functions of digestion, assimilation and vitalization. From any of these impairments in the parents, result delicacy of constitution, defect of temperament, lack of fecundity or tendency to degeneration and disease in the offspring. I might indicate pampering of the parents, too severe in-and-in breeding, exhaustion in any degree from over-work, over-feeding, under-feeding, prolonged lactation in the female, or excessive copulation in the male. It is probable that old Hambletonian, in the days of his greatest popularity, got fewer Dexters from the latter cause. George Wilkes never got remarkable stock during his severe trotting life. And though his trotting life was the severest strain ever imposed upon a sire, yet his constitution and stamina did not yield, as proven by his get after his removal to Kentucky, where he imparted his strength and speed with dams of similar powers. Scientific breeding is not a lottery.

Well kept females among cows and ewes produce more twins and hardier young than those poorly fed.

The excessive feeding of cows to produce marvelous quantities of butter in seven days, or of milk in a year, produces a fevered condition of the organs of digestion and reproduction resulting in broken down constitutions and barrenness. But where high feeding results in obesity, as in swine and shorthorns, the system may be restored to breeding health by lower diet, physic, out-door exercise, and even by working cows under the yoke, by which the fatty, plugged-up ovarian tubes permits again a free channel to the impregnation of the secluded ovaries. Pampering is a most fruitful source of barrenness in domestic animals. It produces a congestion, or inflation of the mucous membrane of the special organs of generation which both general and local treatment too often fail to relieve. These unprofitable conditions can only be ascertained by professional experts, having all the modern methods and means of diagnosis. Bulls suffer from impotency, from pampering and lack of healthful exercise or work, resulting in fatty degeneration of tissues. Impotency in young bulls is caused frequently by excessive service. A single service of the same female is better than many services at any one time of heat. Impotency or barrenness is too often inherited.

Breeding in-and-in has some marked advantages. It enables the breeder to concentrate and perpetuate superlative excellencies, even though obtained by incestuous breeding, but when too long persisted in, the constitution becomes weakened, and the size of the progeny decreases. In-and-in breeding is contrary to nature and to sound judgment and experience. In the wild state, where the indiscriminate herding together of both males and females takes place, the females generally refuse the embrace of their own males; and it is the law of herds of wild animals that the mature and stronger males serve the females. Thus, the perpetuity of the best stock is secured. Mr. Bowdy says that in his large experience in-and-in breeding will undoubtedly insure great certainty as to the product, still, when carried beyond one or two generations it is objectionable; while you secure perfect form, you run the risk of weakening the constitution of the animal you obtain. He further says that the term *in-and-in* breeding is really very indefinite, and points out that while many apply it to near relations, in reality it ought to be applied only to animals having precisely the same blood, as own sister and brother; even these have only half the blood of parents. The male has only half the blood of the dam, and the female only half of the blood of the sire, so this can scarcely be called pure in-and-in breeding, and should, even then, be practiced with thoughtful caution. The gravest and most imminent danger from in-and-in breeding comes from perpetuating defects which, when they once exist in close bred animals, concentrate blood taints that will be magnified as the rays of light passing through a double convex lens which converge the rays of light to a focus, and such breeding centers in the offspring of such couplings the same defects vastly magnified. This is a consummation devoutly to be avoided. Thol's maxim was: breed in-and-in from a bad stock and you commit ruin and devastation—but if good stock be selected you may breed in-and-in as much as you please. He certainly must have bred from a fanciful notion that delicacy and beauty of form were the ultimate wish of the breeder, for the infusion of fresh blood into a family of great excellence is a source of strength and vigor, while the closest consanguinity always tends to degeneration. One or two, or even several incestuous cohabitations of animals may not strike one as injurious when we are blinded by a degree of refinement and beauty in the offspring, but, after all, when we come to study the relative stamina, strength, and constitution of the progeny in a fair, full and unbiased manner, we shall find a surprising degree of degeneration. There may be, however, excusing circumstances, where, for instance, the nearest relations may be bred together to perpetuate a nearly extinct excellence or family. In such cases a risk may be taken rather than to forever lose a coveted quality, for the excellencies of breed is only the sum total of the excellencies of individuals; and though the breed can not be so soon injured by the maltreatment of individuals, yet it ultimately militates against the purity of the flowing stream of the breed; for each animal sends rivulets to the grand stream, and the purity of these assures the purity of the deep and steadily flowing on of the blood of the breed.

I have purposely confined myself to some of the most important matters in breeding, lest I should occupy too much of the space of a JOURNAL so strictly scientific, and I have also purposely drawn from practical experience. I have not in any way attempted to cover the broad ground of breeding domestic animals in the space of a page or two. I trust I have been as fair as brief. By mating the third removes, or by breeding in the line of blood strains, remoter ancestors yield up blood accumulations which have been proven to possess the desired qualities. By this method the fleetest and stoutest running horses have been secured, and the fastest *trotters* bred—in this way the most satisfactory breeding has been practiced in the great establishments of the world.

Next in importance to breeding is feeding. In fact both must be practiced at the same time, as they are inseparably associated. I only give precedence to breeding as in nature the animal must exist before it can be fed. A bad animal may be usefully fed, but a bad animal cannot be profitably bred.

It is common to hurl the mutilating lance of derision at science, but any unprejudiced observer cannot fail to see the beneficent teachings and manifold workings of science in every department of human enterprise. Experience has made intelligent feeders remarkably successful in getting cows to consume enormous quantities of food; but, until very recently in butter tests, science was not importuned to reveal the secrets of perfect feeding for quantity of product. Let us glance at the process of digestion.

The process of digestion is the conversion of solid and insoluble food substances, and of food elements, into minute portions which are thus made more soluble in water or in the digestive fluids, so that these elements of nutrition may pass into the circulation and be most easily appropriated by the animal system for its own sustenance and for the manufacture of products. Until foods are changed in form by being broken up into very small portions, and made soluble by the action of the salivary, gastric, pancreatic and other glandular juices, they cannot be appropriated as food or as products. Nature has provided the means of comminution by means of the teeth and of the solution of food and product elements ordinarily required. But when animals become living machines for converting food into unusual nourishment, and into products of whatever kind of extraordinary quantities, we must resort to science to relieve animals of the extra strain upon their unaided powers for these ulterior designs. We grind coarse hard foods into particles that can be more easily acted upon by the digestive powers, and we must needs convert these ground foods into a soluble state so as to overcome the vital tax upon the animal functions, in order to secure an enormous quantity of product demanded.

The nutritious rations, chemists tell us, are made up of one proportion of protein, eight proportions of carbohydrates, and one of fat. Experiments have proven that about these relative proportions of food elements are the best for the sustenance of the animal system, and for the securing of product. This being the case, great excess of either only clogs the wheels of action of the animal functions. Too much starch, therefore, is worse than too little; too much protein, or too much fat relatively, are hindrances to the proper performance of the animal offices of digestion and assimilation. Physiologists design to convey by the term *assimilation*, the act by which living bodies appropriate and transform into their own substance, matters with which they may be placed in contact. It is the function of nutrition in man; but in our domestic animals it is more than either or both of these; it is the conversion of food elements into products—enormous products of every kind to the fullest possible extent of the animal in the briefest space of time. The age in which we live is only satisfied with the utmost possible results. Or rather, it demands that the utmost results shall be exceeded in all the animal products. The *racer* must excel his own record; the trotter annually beat his best time; the cow must yield a ton of milk, or a thousand pounds of cheese, or six hundred pounds of butter, and all the other animal products must be superlative in quality and quantity. Aid must come from science; cramming down animals the greatest amount of all manner of food and fodder, is not the physiological method of securing the best possible results in either quantity or quality of product. Stuffing is certain to result in deranged digestion which will defeat the grand aim demanded. When breeders and feeders learn that excess of the starch in the ration decreases the digestibility of all other kinds of the fodder elements, they will the better and more certainly secure large amounts of special products. We can no more secure butter from starch, or from fat, or from proteids separately and alone, than we can fatten animals alone on water, though water is an essential property in fattening animals. The excrements of animals over-fed on starchy foods, will show undigested starch, by the aid of the microscope, or by the iodine test. It is this wasteful kind of feeding that detracts from the receipts from the dairy, the farm, and from nearly all of the products of domestic animals; and especially is there a great waste of feed in old horses and hard worked city horses. Could the amount of waste in dollars be estimated in the United States it would show a fearful prodigality that would be astonishing, for it would reach millions annually. W. Mathieu Williams aptly says: "Man alters the position of physical things in such-wise, that the forces of nature shall operate upon them, and produce the changes or other results that he requires." This is said of the man who has become conversant with the laws of nature, and not of him who plunges in without thought, reflection or knowledge. It might justly be said of the average farmer that he is the most wasteful of all men, for from his abundance he saves so little.

The following table contains a summary of the results of experiments by Henneberg & Strohmann, E. Schulze & Moreker, Strohmann & Wolff, compiled from Wolff. The first column contains the name of the experimenter; the second, the amount of starch fed, expressed in per cent. of the dry matter of the remaining fodder; the third shows the character of the other fodder; the fourth and fifth express the decrease in the digestibility of the protein and crude fiber in per cent. of the quantity of each which was digested when the starch was withheld. The above and the tables I quote from the valuable compilation of Armsby. And I specially request a careful study of the tables, in order that we may fully ascertain the astonishing facts which form the foundation of the physiological principles of rational feeding. It is also a settled fact that excess of starch or sugar diminishes the digestibility of portein and fiber, indicating clearly the necessity of observing and respecting a medium nutritive ratio in feeding animals for whatsoever products.

| No. | Authority. | Starch in Per Cent. of other Fodder. | Fodder exclusive of Starch. | Decrease Indigestion of | |
|---|---|---|---|---|---|
| | | | | Protein Per Cent. | Crude Fiber Per Cent. |
| 1 | Henneberg and Strohmann...... | 15 | Clover hay,straw,and beans. | 7 | 6 |
| 2 | .............................. | 18 | .............................. | 11 | 7 |
| 3 | Henneberg and Strohmann ..... | 29 | .............................. | 21 | 15 |
| 4 | Henneberg and Strohmann ..... | 9 | .............................. | 3 | 4 |
| 5 | Henneberg and Strohmann...... | 9 | Same with more beans ........ | 4 | 2 |
| 6 | Schulze and Morecker.............. | 25 | Hay.............................. | 41 | 10 |
| 7 | Schulze and Morecker.............. | 25 | Hay and beans.................. | 20 | 13 |
| 8 | Strohmann........... | 15 | Hay .............................. | 12 | 12 |
| 9 | Strohmann............ | 69 | Hay .............................. | 44 | 7 |
| 10 | Strohmann............ | 15 | Hay and oil cake ............... | 9 | 10 |
| 11 | Wolff(experiments on hays).......... | 15 | Barley.............................. | 0 | .............. |
| 12 | Wolff(experiments on hays).......... | 31 | Barley.............................. | 11 | .............. |

Two things are shown by this table. First, the greater the amount of starch which is added to a ration, the more is the digestibility of the protein and crude fiber decreased, e. g. in experiments, 1, 2 and 3, or 8 and 9; second, the richer the original ration in protein, the less is the depression caused by a given quantity of starch, e. g. experiments 6 and 7.

Strohmann's formula is here given, and though it is from inductions, and dependent upon a large number, doubtless, of accurate observations, yet these formulas are not based upon a mathematical certainty, but still possess much value.

"$P' = \frac{P}{1 + \frac{1}{8} P} S.$ In which $P'$ represents the digestible protein, $P$ the total crude protein, and $S$ the total non-nitrogenous matters of the ration, including fat."

I quote the following from Armsby:

"Indeed a too wide nutritive ratio may cause more waste than a too narrow one. In the former case the protein consumption is * * * needlessly increased, but the nitrogen of this protein is excreted in the urine, and has its value in the manure. In the second case, a too-wide nutritive ratio also causes a waste of protein by decreasing its digestibility, but it also causes some of the starch to pass through the body without being put to any use, while as manure the latter is absolutely valueless, containing only elements of which the atmosphere offers an inexhaustable supply to plants." Animals throw off carbonic acid, while growing plants absorb it through their myriad mouths located in every leaf.

I find so much that is significant and pertinent to the subject under consideration in Dr. G. C. Caldwell's article to the New York *Weekly Tribune*, entitled *Protein Rich Food For Pigs —Three Strong Reasons for its Use*, that I would quote it entire, had I the space, but must be content with the briefest possible statement of conclusions in it. He says: "Professor J. W. Sanborn reports that *feed rich in protein makes more lean meat and less fat: that,* 100 *lbs. of ship-stuff will go as far as* 108 *lbs. of corn meal in respect to economy in production of increase in weight; and that feed richer in protein makes richer manure.*" The above was in reference to feeding pigs, but applies equally well in the feeding of other animals.

Cooking food at a low temperature is quite inexpensive, and like grinding, but still better, it predigests the elements of foods so as to relieve the unnecessarily taxed functions of feeding animals, as well as those who perform fast or severe work. Malt also possesses large proportions of the *amines, amides,* and *amids-acids,* which predigest the starchy elements of foods. These have a close chemical resemblance to ammonia which plays so prominent a part in the animal and vegetable world. Malting of the grains not only enrich the poorer fodders by adding albuminoids, and in promoting the conversion of starchy foods into soluble and thus readily digestible substances, but they also promote and stimulate the digestibility of all foods and fodders. It is by the use of rich proteids possessing these delicate and potent qualities that enormous products in milk, butter, cheese, meat, or labor are procurable by scientific feeding without the risk of injury to the animals fed. Let us look at one single instance of the potentiality of these *amids-acids* and nitrogenous organic substances upon the possibilities of feeding for products. To a thick pudding of oatmeal porridge heated to a temperature of 150° Fah., stir in one-sixth quantity of perfectly dry *malt flour*, and a marvelous change soon takes place. Instead of becoming dryer and harder, it acts in consistency like adding water, it converts the thick pudding into a thin, sweet, and easily digested porridge. This results from the powerful action of the *diastase of malt.*

